# Supplementary material for: Sequence-to-sequence translation from mass spectra to peptides with a transformer model
Source: Nat Commun. 2024 Jul 30;15:6427. doi: 10.1038/s41467-024-49731-x (PMC11289372; doi:10.1038/s41467-024-49731-x)
Supplement: Supplementary file 1 — Supplementary information [file 41467_2024_49731_MOESM1_ESM.pdf]

Supplement to "Sequence-to-sequence translation from mass spectra to peptides with a transformer model"

Melih Yilmaz*[1], William E. Fondrie*[2], Wout Bittremieux*[3], Carlo F. Melendez[4], Rowan Nelson[4], Varun Ananth[1], Sewoong Oh[1], and William Stafford Noble†[4,1]

[1]Paul G. Allen School of Computer Science and Engineering, University of Washington
[2]Talus Bioscience
[3]Department of Computer Science, University of Antwerp
[4]Department of Genome Sciences, University of Washington

*Equal contributions
†Corresponding author: willilam-noble@uw.edu

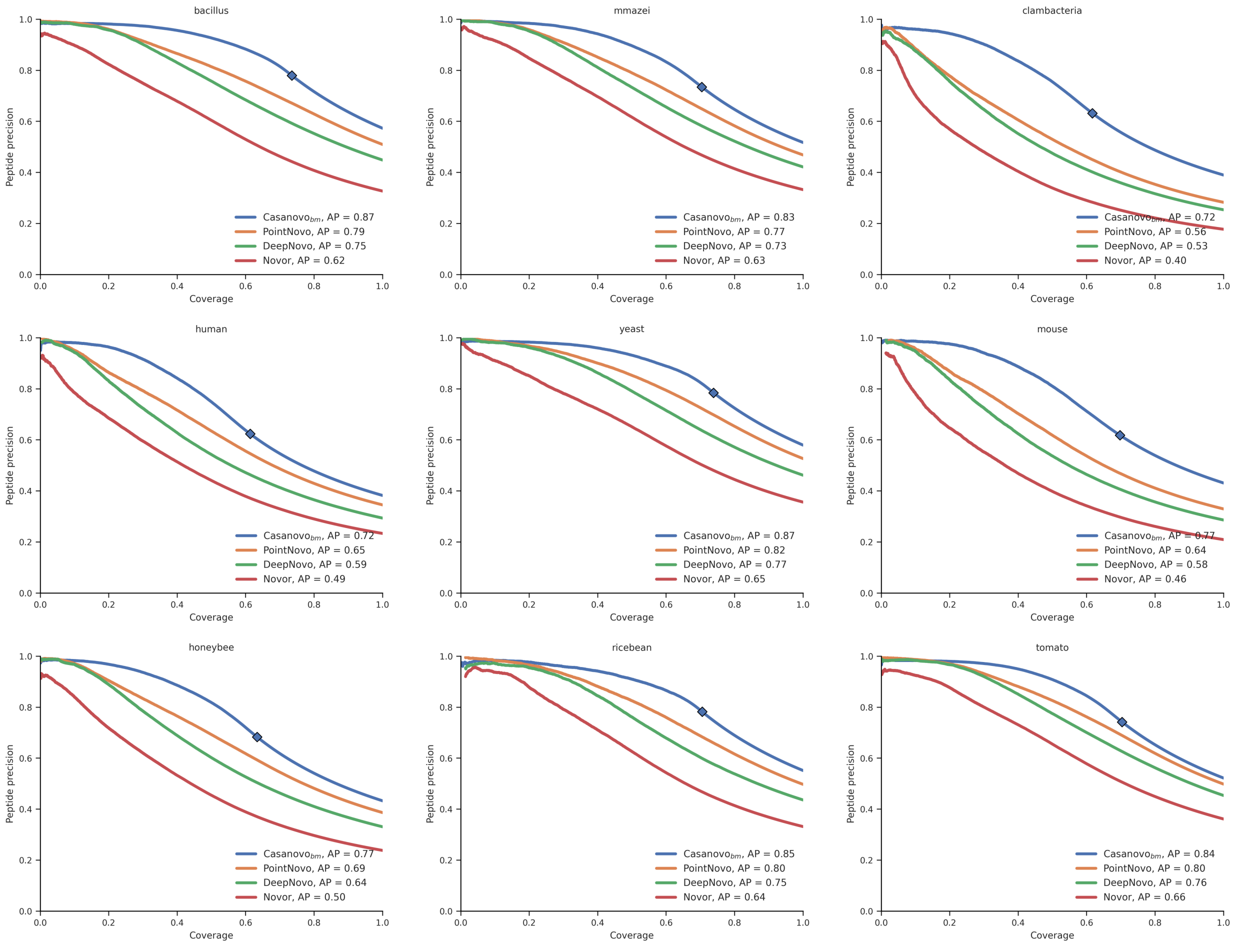

Figure S1: **Casanovo$_{bm}$ outperforms Novor, DeepNovo, and PointNovo on the original nine species benchmark.** Each panel evaluates the peptide-level performance on the held-out species in the nine species benchmark. For Casanovo$_{bm}$ all peptides that pass the precursor m/z filter are ranked above peptides that do not pass the filter, and the boundary is indicated by a diamond on the curve.

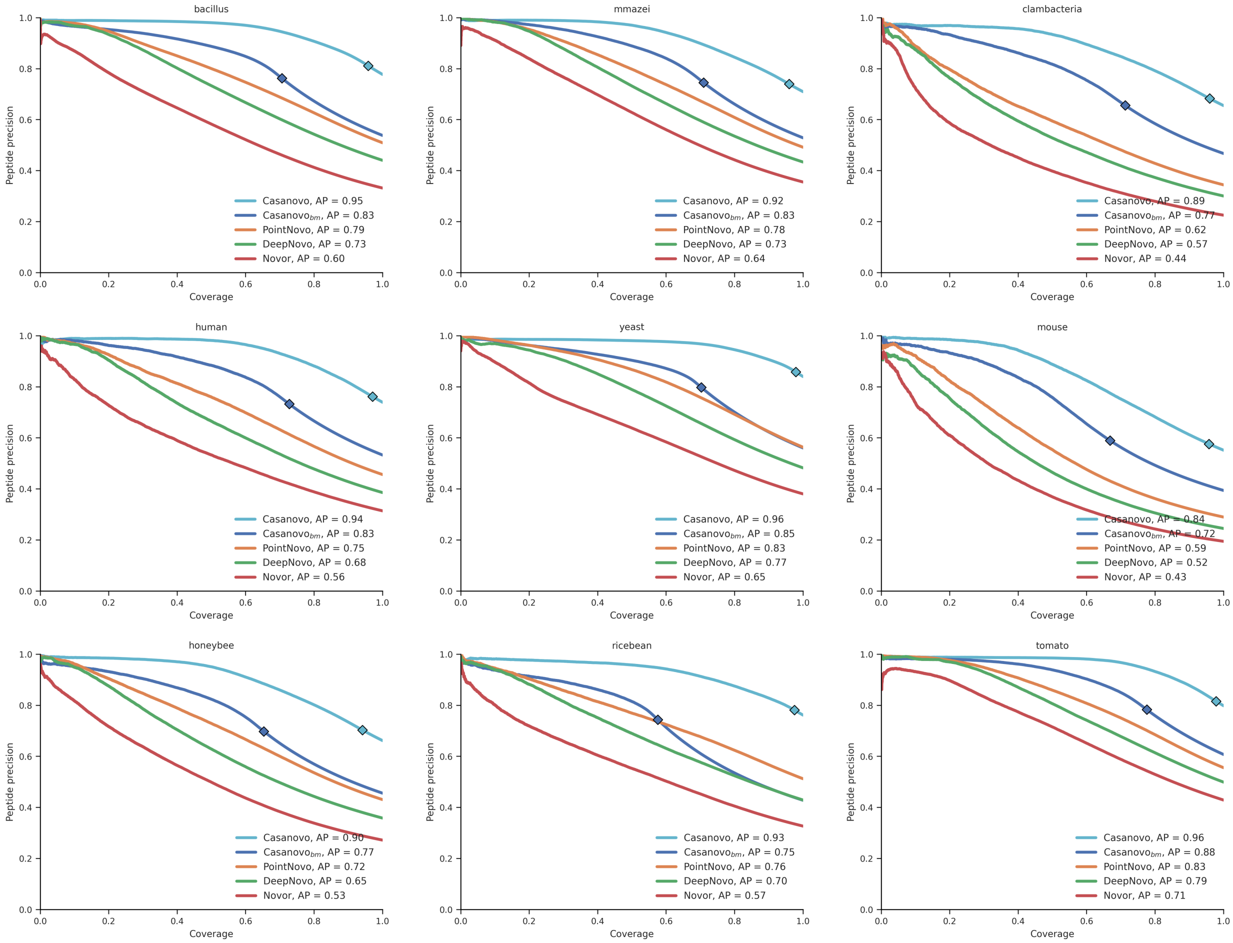

Figure S2: **Casanovo outperforms Novor, DeepNovo, PointNovo, and Casanovo$_{bm}$ on the revised nine-species benchmark** Each panel evaluates the peptide-level performance on the held-out species in the nine species benchmark. For Casanovo and Casanovo$_{bm}$ all peptides that pass the precursor m/z filter are ranked above peptides that do not pass the filter, and the boundary is indicated by a diamond on each curve.

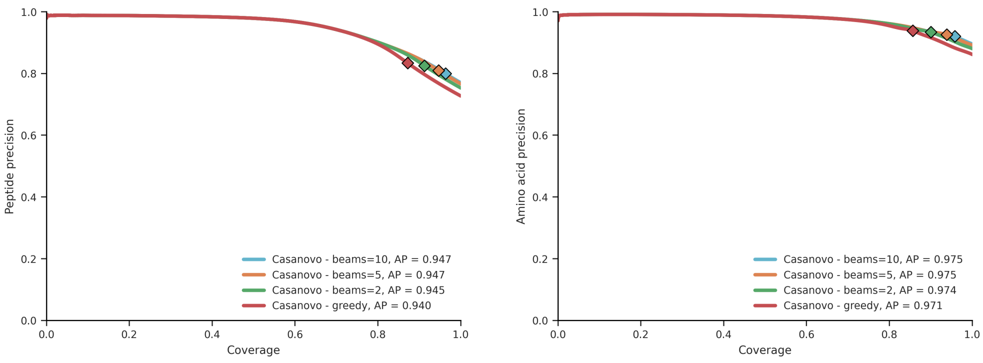

A B

Figure S3: **Comparison of greedy and beam-search decoding.** (A) The plot shows precision-coverage curves for the Casanovo model, using either greedy decoding or beam-search decoding with different number of beams. The revised 9-species benchmark was used for this analysis. (B) Similar to panel (A), but showing amino acid-level precision and coverage for the same data set.

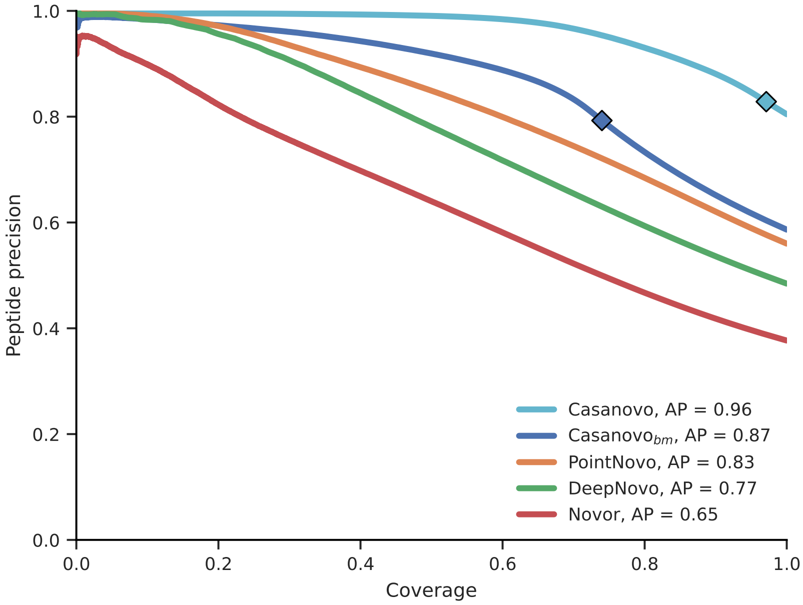

Figure S4: **Casanovo outperforms Novor, DeepNovo, PointNovo, and Casanovo$_{bm}$ on the revised nine-species benchmark, even when PTMs unavailable to DeepNovo or Novor are eliminated.** The figure plots peptide-level precision as a function of coverage for all species in the nine-species benchmark for Casanovo, DeepNovo and Novor. Casanovo maintains higher peptide-level precision across the full coverage range over Novor, DeepNovo, PointNovo, and Casanovo$_{bm}$ on the aggregated, revised nine-species benchmark. MS/MS spectra associated with modifications that cannot be detected by DeepNovo and Novor are eliminated.

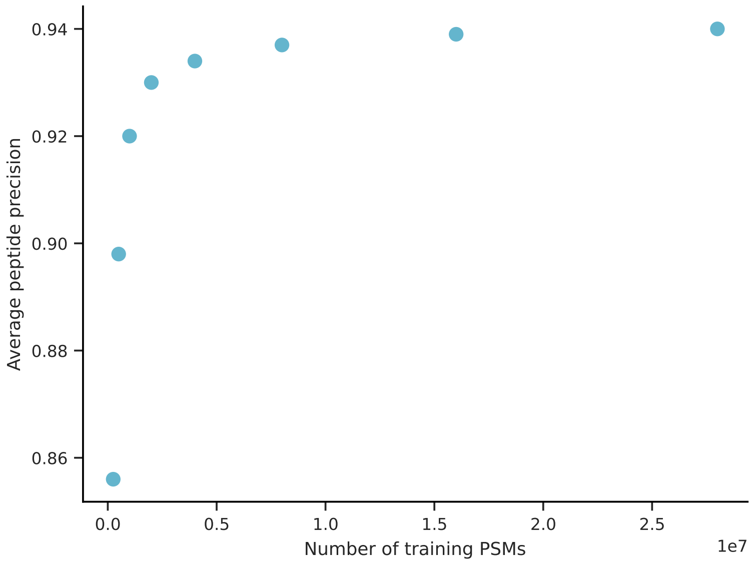

Figure S5: **Casanovo performance on the 9-species benchmark improves with more training data.** Each point corresponds to a Casanovo model trained on one of the nested subsets of MassIVE-KB, ranging from 250,000 spectra to the full dataset of 28 million spectra. Average precision is reported on the revised 9-species benchmark.

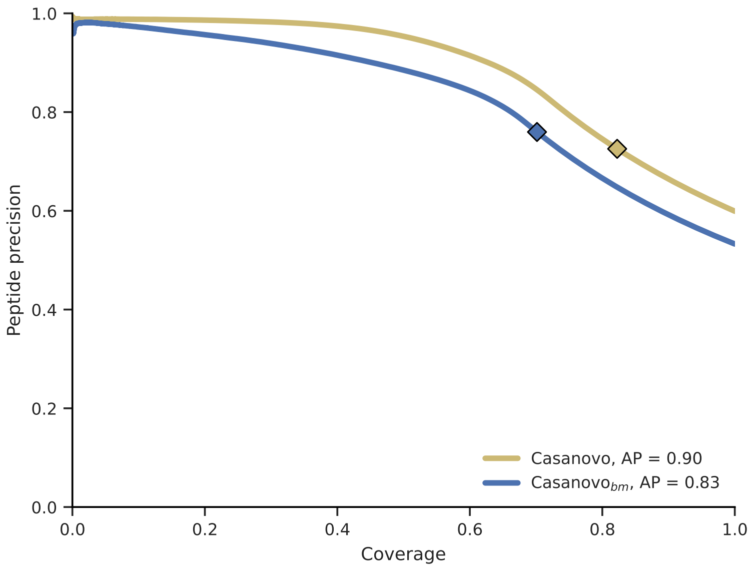

Figure S6: **Comparison of Casanovo models trained on the 9-species benchmark and MassIVE-KB.** The figure plots, for each model, precision on the revised 9-species benchmark as a function of coverage. The training sets for Casanovo$_{bm}$ and MassIVE-KB trained Casanovo model contain 246,713 and 239,697 distinct peptides, respectively.

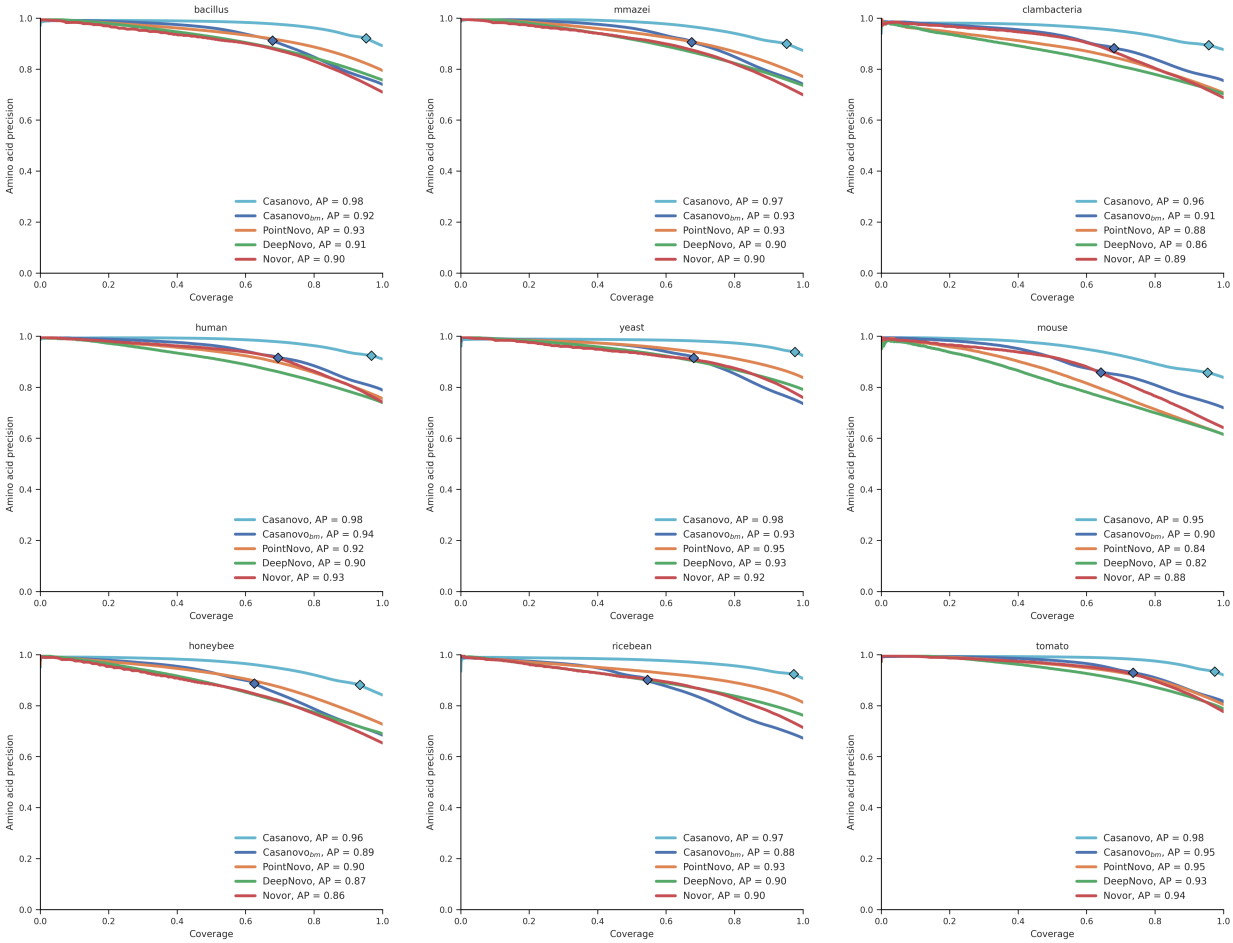

Figure S7: **Casanovo outperforms Novor, DeepNovo, PointNovo, and Casanovo$_{bm}$ at the amino acid-level on the nine-species benchmark** Each panel evaluates the amino acid-level performance on the held-out species in the nine species benchmark. For Casanovo and Casanovo$_{bm}$ all peptides that pass the precursor m/z filter are ranked above peptides that do not pass the filter, and the boundary is indicated by a diamond on each curve.

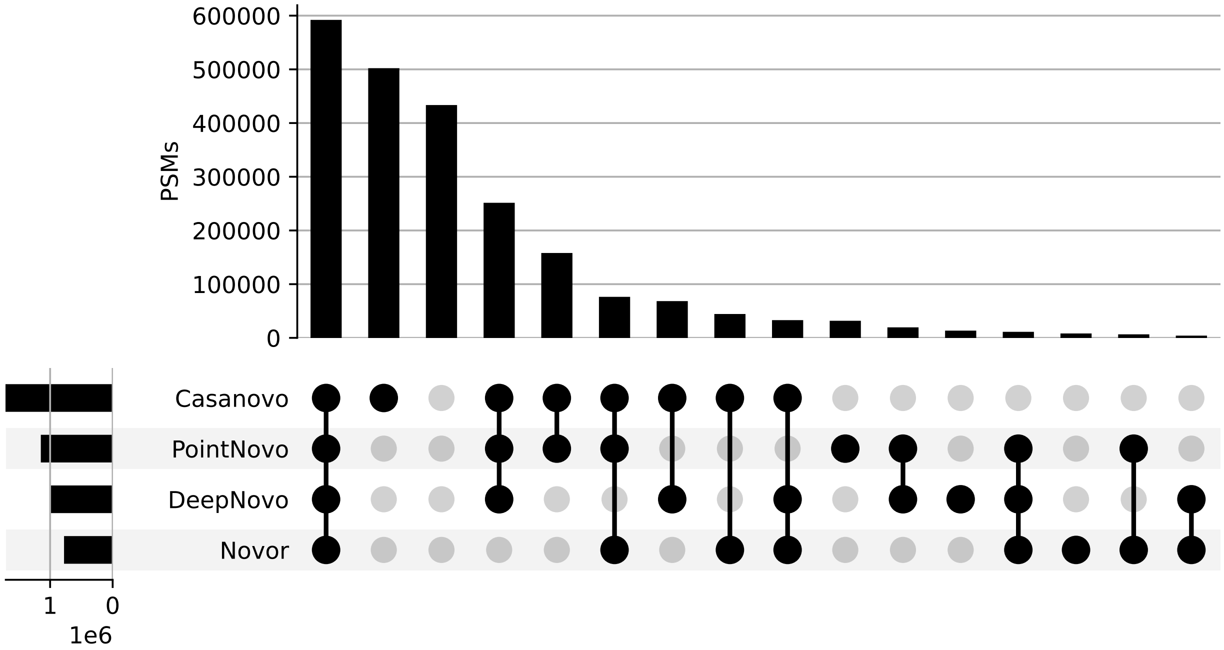

Figure S8: **Casanovo expands the number of correct PSMs identified by its competitors on the nine-species benchmark.** The plot shows the overlap in peptide predictions between Casanovo and three competing *de novo* sequencing methods for the nine-species benchmark dataset. For each subset of PSMs, black circles denote whether the corresponding method is correct. Horizontal bars indicate the total number of correct PSMs for each method.

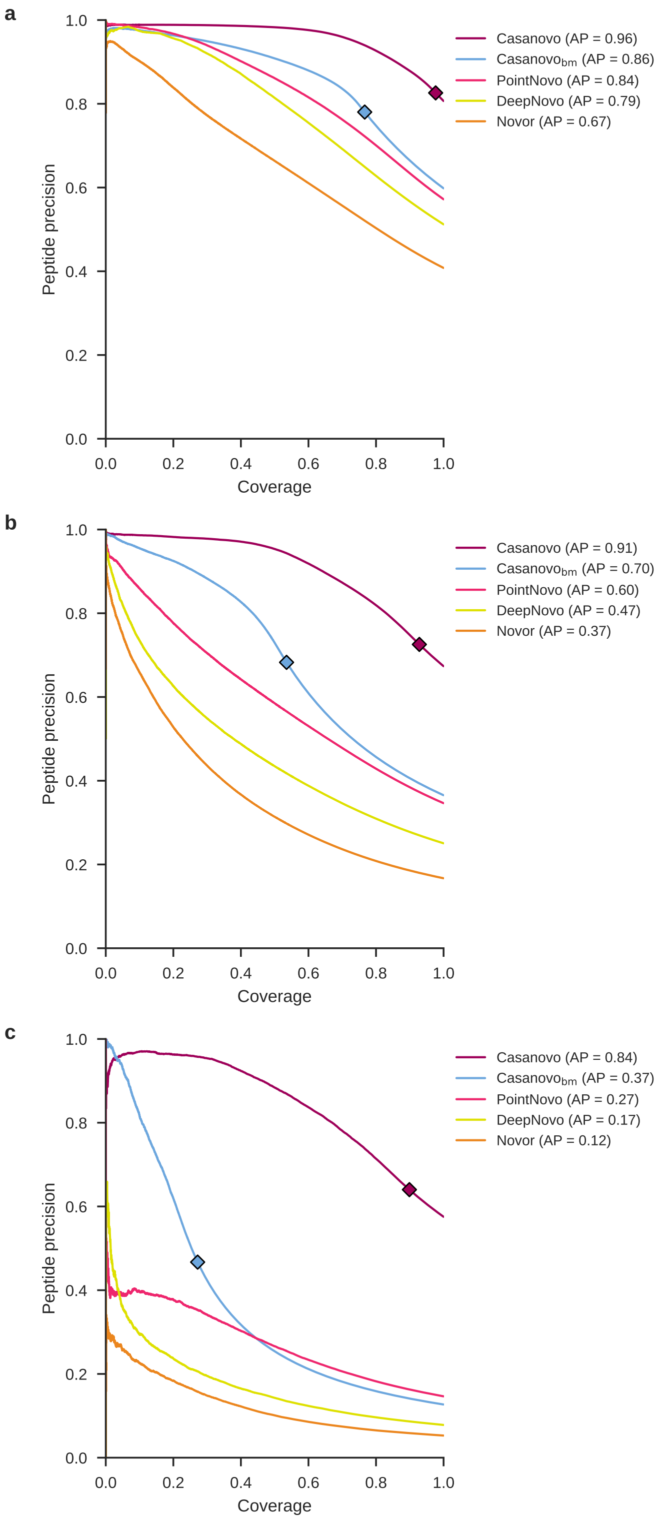

Figure S9: **Breakdown of *de novo* sequencing performance by charge state.** Plots show peptide precision-coverage curves for subsets of the revised nine-species benchmark, grouped by charge state where panels correspond to spectra with (A) 2+ charge, (B) 3+ charge, (C) 4+ or higher charge.

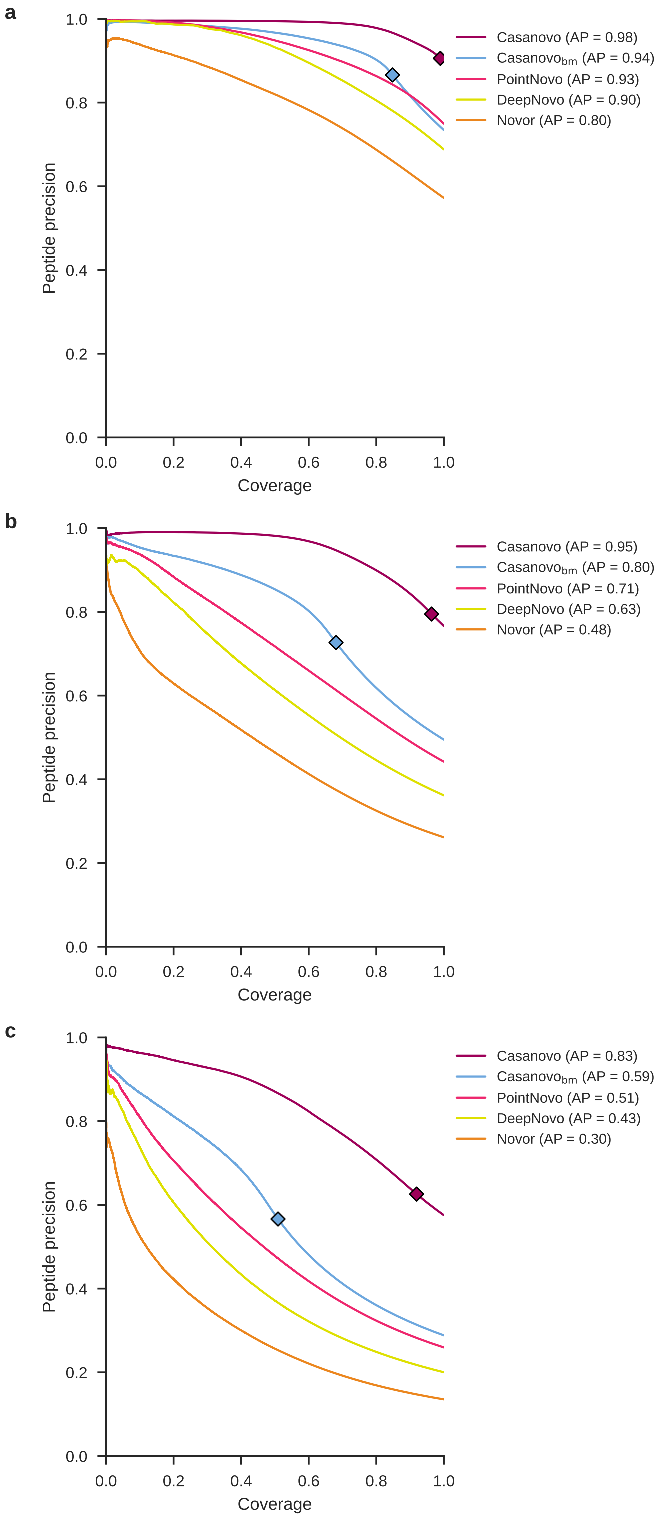

Figure S10: **Breakdown of *de novo* sequencing performance by peptide length.** Plots show peptide precision-coverage curves for subsets of the revised nine-species benchmark grouped by the length of database search assigned peptides where panels correspond to peptides with (A) fewer than 13 amino acids (B) between 13 and 18 amino acids, (C) greater than 18 amino acids.

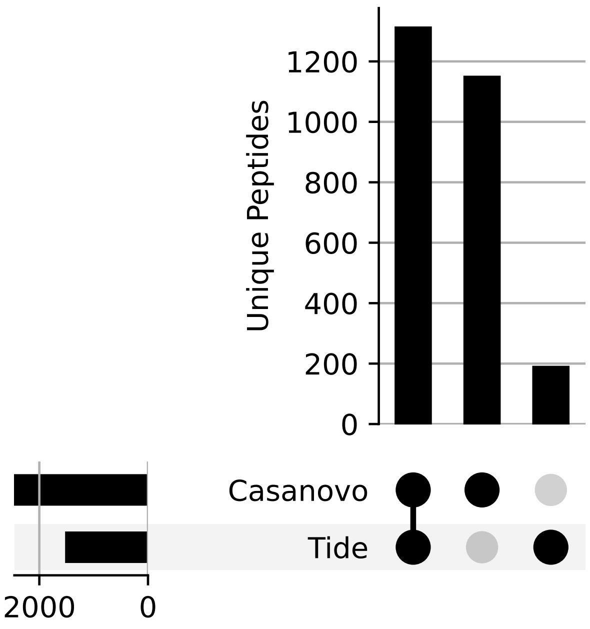

Figure S11: **Casanovo identifies a greater number of immunopeptides than Tide database search.** The plot shows the overlap between unique peptides assigned by Casanovo that match to the human proteome and by Tide at 1% FDR for the immunopeptidomics dataset.

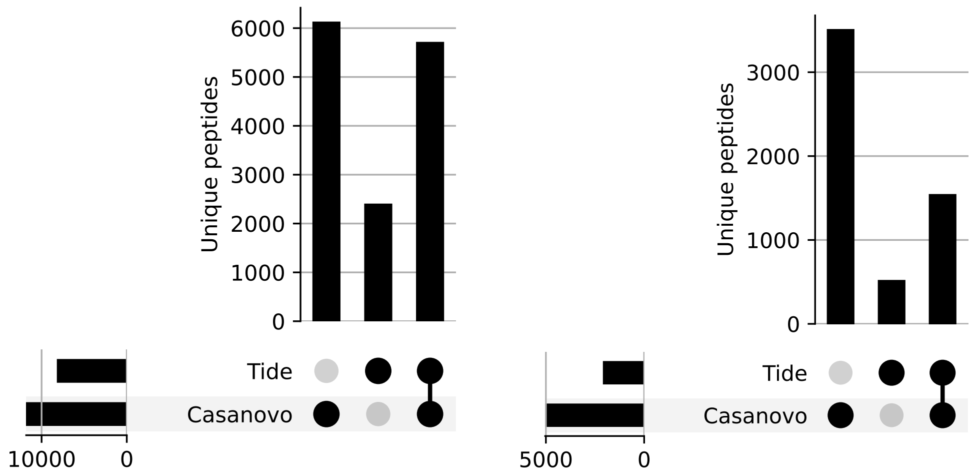

A B

Figure S12: **Casanovo detects a substantial number of additional peptides compared to Tide database search in metaproteomics samples.** (A) The plot shows the overlap between unique peptides assigned by Casanovo at 1% error rate and Tide at 1% FDR when respective metapeptide databases are used for the Bering Sea and the Chukchi Sea datasets. (B) Similar to panel (A), but the non-redundant environment database is used for searching and error rate control.

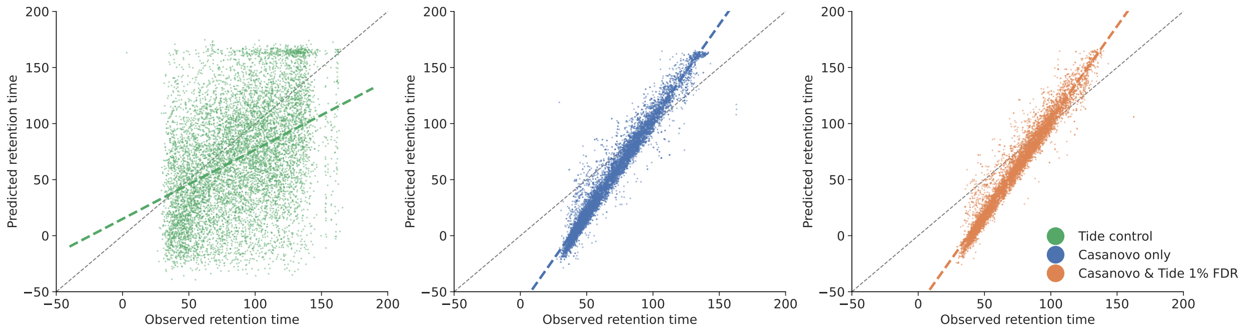

Figure S13: **Confident Casanovo assignments have retention times that are highly correlated with Prosit predictions.** We compared the observed retention times against Prosit-predicted retention times for three groups of peptides: (1) peptides predicted only by Casanovo that match to the relevant database at 1% error rate, (2) peptides identified by both Casanovo and database search with a 1% FDR threshold, and (3) peptides identified by database search with FDR >10%. The dashed line in each plot represents the ordinary least squares linear regression.

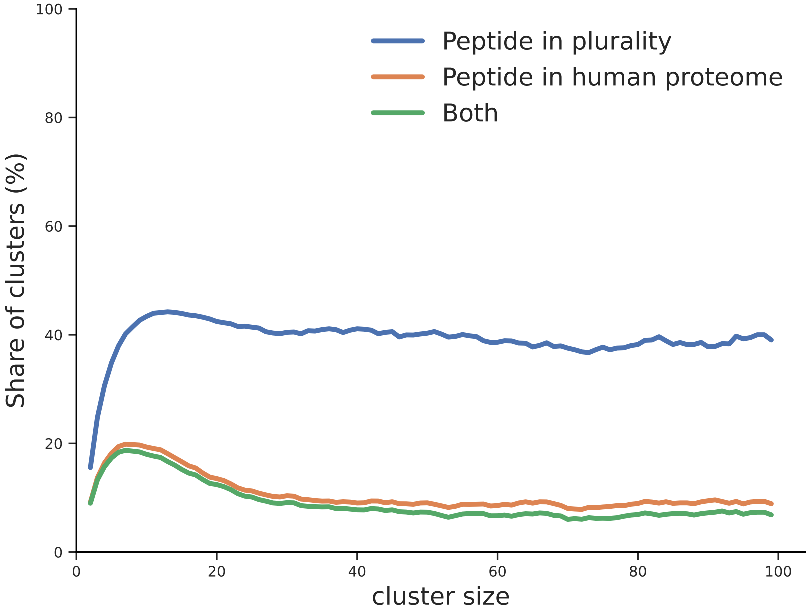

Figure S14: **Casanovo assigns new peptides to dark matter clusters of various sizes.** We evaluated the fraction of clusters which have a Casanovo peptide in plurality, have a unique Casanovo peptide matching to human proteome or satisfy both criteria among all previously unidentified clusters larger than a given size.

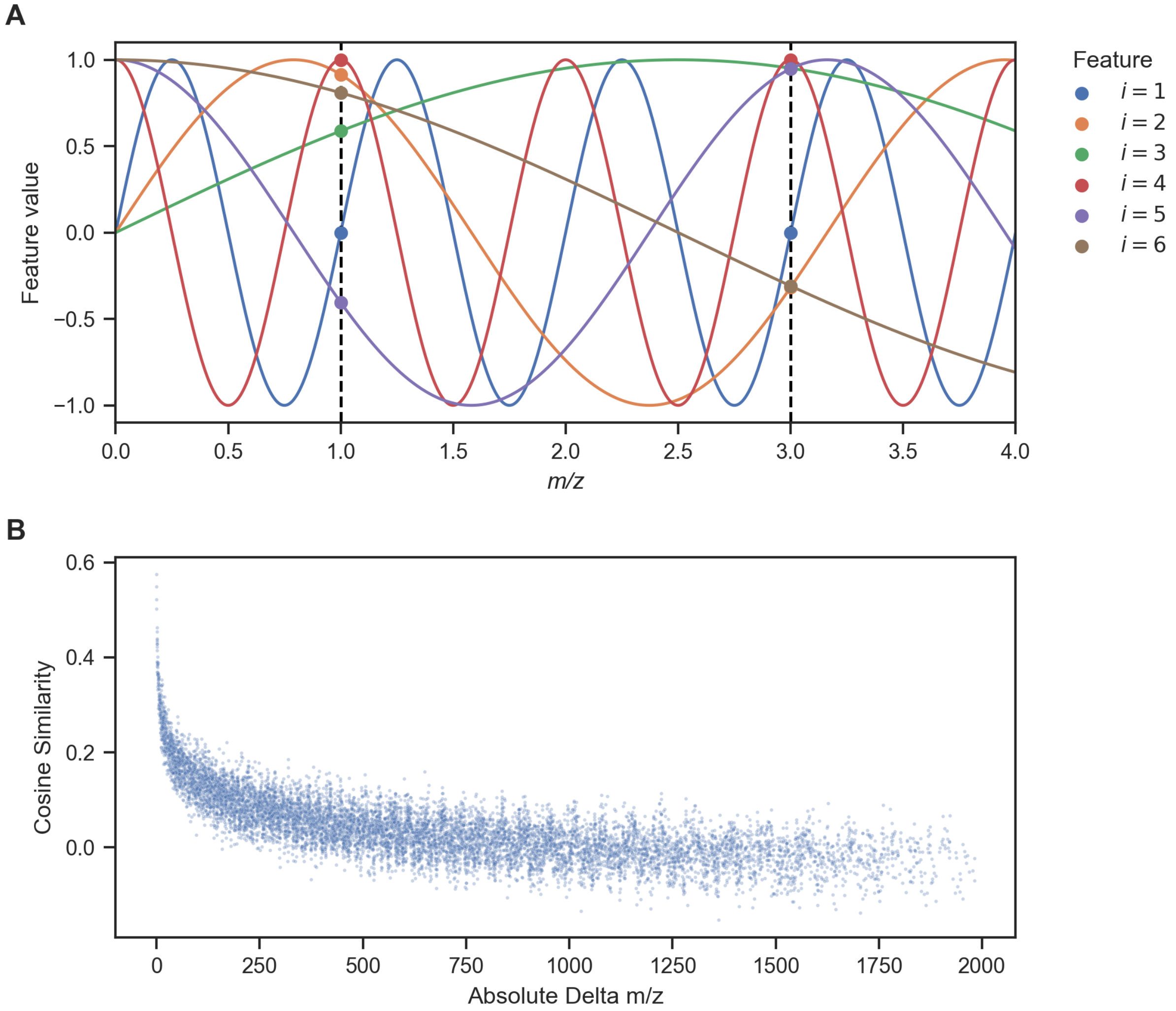

Figure S15: **Sinusoidal encodings represent m/z distance between peaks in a mass spectrum.** **(A)** The m/z value of each peak is encoded from a progression of sinusoids defined by a minimum and maximum wavelength. In this example, a 6-dimensional embedding ($d = 6$) of m/z 1.0 and m/z 3 is created from sinusoids ranging from a wavelength of m/z 1 ($\lambda_{\text{min}} = 1$) to 10 ($\lambda_{\text{max}} = 10$)to demonstrate how the encoding is performed. **(B)** The Casanovo sinusoidal embeddings are 512-dimensional ($d = 512$) and created from sinusoids ranging from m/z 0.0001 ($\lambda_{\text{min}} = 0.0001$) to 10,000 ($\lambda_{\text{min}} = 10,000$). The utility of these embeddings lies in their preservation of m/z distance in their embedded space. Here, we sample 10,000 pairs of m/z values between m/z 0 and 2000. The cosine similarity between these embeddings is negatively correlated with the original distance between m/z values.

	MassIVE-KB count	MassIVE-KB selected	PROSPECT count	PROSPECT selected
A	24910	24910	540777	25090
C	11473	11473	90772	38527
D	44654	44654	267716	5346
E	42977	42977	603469	7023
F	38183	38183	884261	11817
G	21640	21640	344670	28360
H	166398	50000	357268	0
I	12984	12984	437698	37016
K	16289160	50000	960538	0
L	50002	50000	2264210	0
M	19405	19405	388750	30595
N	40867	40867	294550	9133
P	9685	9685	114504	40315
Q	33572	33572	542063	16428
R	13589301	50000	1135502	0
S	28389	28389	457519	21611
T	15845	15845	443535	34155
V	26011	26011	774624	23989
W	4261	4261	341456	45739
Y	35180	35180	1368308	14820
Total	30504897	610036	12612190	389964

Table S1: **Creating a non-enzymatic dataset by sampling from PROSPECT and MassIVE-KB.** PROSPECT was first downsampled to include at most 100 PSMs per peptide sequence. MassIVE-KB and PROSPECT were then segregated by C-terminal amino acid, and we randomly selected from each category from MassIVE-KB, supplementing as necessary from PROSPECT to obtain 50,000 PSMs per terminal amino acid.

PRIDE	Species	Uniprot	Files	Spectra	PSMs	Peptides	precursor	fragment
PXD005025	*Vigna mungo*	UP000087766	24	932848	108514	12001	20	0.05
PXD004948	*Mus musculus*	UP000000589	13	306786	25541	5899	10	0.05
PXD004325	*Methanosarcina mazei*	UP000033058	72	3728183	267333	15925	10	0.05
PXD004565	*Bacillus subtilis*	UP000001570	106	4336428	1358337	30786	30	0.05
PXD004536	*Candidatus endoloripes*	UP000094849	11	2272023	82290	8392	20	0.05
PXD004947	*Solanum lycopersicum*	UP000004994	60	603506	178413	49745	15	0.05
PXD003868	*Saccharomyces-cerevisiae*	UP000002311	27	1477397	585593	19720	20	0.05
PXD004467	*Apis mellifera*	UP000005203	17	823169	194281	21559	20	0.05
PXD004424	*H. sapiens*	UP000005640	26	684821	44604	11289	20	0.02
Total			343	15,165,161	2,844,906	175,316		

Table S2: **The nine-species benchmark.** The final two columns specify the precursor window size (in ppm) and fragment bin size (in Da) used in the database search step. No reference proteome is available for *Vigna mungo*, so the proteome for the closely related species *Vigna radiata* was used instead.

In human proteome	In Casanovo peptide	BLOSUM score	Count
L	V	1	749
V	L	1	650
E	Q	2	467
N	D	1	437
R	K	2	371
E	H	0	343
E	D	2	338
L	K	-2	314
L	M	2	304
L	F	0	301

Table S3: **The most common amino acid swaps have positive BLOSUM scores.** We found that the top ten most common single amino acid substitutions that can be explanied with a single nucleotide polymorphism detected by Casanovo are enriched for positive BLOSUM scores.
